# Intersectionality of inequalities in revascularization and outcomes for acute coronary syndrome in England: nationwide linked cohort study

**DOI:** 10.1093/ehjqcco/qcae112

**Published:** 2025-01-30

**Authors:** Marius Roman, Ann Cheng, Florence Y Lai, Hardeep Aujla, Julie Sanders, Jeremy Dearling, Sarah Murray, Mahmoud Loubani, Vijay Kunadian, Chris Gale, Gavin J Murphy

**Affiliations:** Department of Cardiovascular Sciences and National Institute for Health Research Leicester Biomedical Research Unit in Cardiovascular Medicine, University of Leicester, Glenfield Hospital, Groby Rd, Leicester LE3 9QP, UK; Department of Cardiovascular Sciences and National Institute for Health Research Leicester Biomedical Research Unit in Cardiovascular Medicine, University of Leicester, Glenfield Hospital, Groby Rd, Leicester LE3 9QP, UK; Department of Cardiovascular Sciences and National Institute for Health Research Leicester Biomedical Research Unit in Cardiovascular Medicine, University of Leicester, Glenfield Hospital, Groby Rd, Leicester LE3 9QP, UK; Department of Cardiovascular Sciences and National Institute for Health Research Leicester Biomedical Research Unit in Cardiovascular Medicine, University of Leicester, Glenfield Hospital, Groby Rd, Leicester LE3 9QP, UK; Faculty of Nursing, Midwifery & Palliative Care, King's College, London WC2R 2LS, UK; St Bartholomew's Hospital, Barts Health NHS Trust, London, UK; National Cardiac Surgery Patient and Public Involvement (PPI) Group, University of Leicester, Glenfield Hospital, Groby Road, Leicester LE3 9QP, UK; National Cardiac Surgery Patient and Public Involvement (PPI) Group, University of Leicester, Glenfield Hospital, Groby Road, Leicester LE3 9QP, UK; Department of Cardiothoracic Surgery, Castle Hill Hospital, Cottingham HU16 5JQ, UK; Cardiothoracic Centre, Freeman Hospital, Newcastle upon Tyne NHS Foundation Trust, Newcastle upon Tyne NE7 7DN, UK; Leeds Institute of Cardiovascular and Metabolic Medicine, University of Leeds, Leeds LS2 9JT, UK; Leeds Institute for Data Analytics, University of Leeds, Leeds LS2 9NL, UK; Department of Cardiology, Leeds Teaching Hospitals NHS Trust, Leeds Leeds LS1 3EX, UK; Department of Cardiovascular Sciences and National Institute for Health Research Leicester Biomedical Research Unit in Cardiovascular Medicine, University of Leicester, Glenfield Hospital, Groby Rd, Leicester LE3 9QP, UK

**Keywords:** Acute coronary syndrome, Hospital episode statistics, Inequalities

## Abstract

**Background:**

Inequalities in access to care for women, people of non-white ethnicity, who live in areas of social deprivation, and with multiple long-term conditions lead to inequity of outcomes. We investigated the intersectionality of these causes of health inequality on coronary revascularization and clinical outcomes for admissions with acute coronary syndrome (ACS).

**Methods and results:**

We included hospital admissions in England for types of ACS from April 2015 to April 2018 and linked Hospital Episode Statistics to the Office for National Statistics mortality data. The primary outcome was time to all-cause mortality. Time-to-event analyses examined the associations of sex, ethnicity, and socioeconomic deprivation with revascularization. Of 428 700 admissions with ACS, 212 015 (48.8%) received revascularization within 30 days. Women, black ethnicity, multimorbid, and frail patients were less likely to undergo revascularization. South Asian ethnicities had higher [hazard ratio (HR) = 1.15, 95% confidence interval (CI) 1.14–1.17] revascularization rates and comparable risk-adjusted survival but higher re-admission rates when compared to other ethnic groups. Women had higher 1-year all-cause [25.5% vs. 14.7%—ST-elevation myocardial infarction (STEMI); 24.9% vs. 18.7%—non-ST-elevation myocardial infarction (NSTEMI)] and cardiovascular (22.6% vs. 13.2%—STEMI; 20.3% vs. 15.6%—NSTEMI) mortality than men. After adjusting for confounders, women had a lower all-cause mortality when compared to men.

**Discussion:**

Outcomes attributed to women and people of South Asian ethnicity may be attributable to age, comorbidity and frailty at presentation. Black ethnicity, geography, and social deprivation may be sources of inequality. These findings highlight the unmet need and may provide potential targets for interventions that address inequalities.

## Graphical Abstract

**Graphical Abstract:**
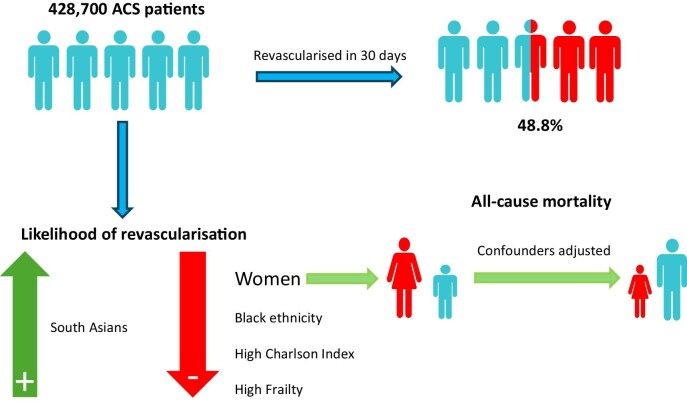

Key Learning Points

- In routinely collected health data of 428 700 patients presenting with ACS, 48.8% received revascularisation.
- The likelihood of revascularisation was higher in South Asian ethnicities but lower in Women, Black ethnicities, high Charlson Index and high frailty-risk patients.
- Women had higher all-cause and cardiovascular mortality but were lower after adjusting for confounders when compared to men.
- Interventions are needed to address inequalities and confounders in people presenting with ACS.

## Introduction

Acute coronary syndrome (ACS) is a leading cause of death worldwide, accounting for over 17 million deaths every year globally.^1^ In the UK, ACS results in over 100 000 admissions to hospitals and coronary heart disease causes around 68 000 deaths per year.^2^ Revascularization is an effective treatment for many people who present with ACS, where it has been shown to reduce deaths, rehospitalization, and other major cardiovascular events (MACEs).^3–5^ Yet, disparities in care for ACS are well described, commonplace across the globe, and associated with patient factors and social determinants.^6,7^

Inequalities in access to care disproportionately affect women, people of non-white ethnicity, people who live in areas of social deprivation, and people with multiple long-term conditions (MLTC).^3,8,9^ These inequalities result in inequity of outcomes.^10^

Risk factors for inequity often co-exist. For example, women, who represent over a third of people presenting with ACS, experience lower crude revascularization rates and worse survival compared to males.^9,11–14^ However, females are also older and have more MLTC than men at presentation with ACS.^15^ Potentially, interventions addressing inequality in the care of women with ACS are likely to be impacted by other co-existent risk factors. For this reason, interventions must address the intersectionality of risk factors for inequality if they are to be most effective.

Previous studies have described variations in practice or outcomes, and most analyses are based on convenience sampling, regions of hospitals, or subgroups defined by age or acute myocardial infarction phenotype [either ST-elevation myocardial infarction (STEMI) or non-STEMI (NSTEMI)].^13,16^

In England, all admissions to all hospitals are recorded for administrative purposes. Entries are classified according to the International Classification of Diseases 10th Revision (ICD-10) classification. Similarly, all deaths in England are recorded, and because each person in England has a unique identity (National Health Service number), their data may be identified and linked between nationwide registries and health databases. This results in high-quality data and comprehensive insights into access, delivery, and care-related outcomes. We used HES linked to Office for National Statistics (ONS) data to investigate the intersectionality of common causes of health inequality regarding access to revascularization and clinical outcomes for people admitted to hospitals with ACS in England. We specifically studied the ACS type, geography, sex, ethnicity, socioeconomic deprivation, age, MLTC, and frailty.

## Methods

### Data sources

We performed a retrospective observational cohort study of hospital admissions with ACS using routine data collected in the Health Episode Statistics^17^ dataset. The data covered all NHS hospital admissions or independent providers funded by NHS,^18^ and it was linked with the ONS mortality data between 1st of April 2015 and 1st of April 2018. NHS Digital provided the HES and the HES–ONS-linked mortality data. This linkage allowed the identification of ACS admissions that resulted in death and separation from death from any other cause. Each record contained demographics (age, sex, ethnicity, area of residence, and socioeconomic deprivation index), administrative (admission and discharge dates, admission method, discharge destination, etc.), and clinical details (diagnoses and procedures performed). Diagnoses were coded based on the ICD-10 codes, and procedures were coded by the OPCS Classification of Interventions and Procedures (OPCS-4). This is the procedural classification used by hospitals within National Health Service (NHS) hospitals of NHS England, NHS Scotland, NHS Wales, and Health and Social Care in Northern Ireland.

### Population

The study included all adult patients (aged ≥18 years) who had at least one hospital admission with an ACS diagnosis (ICD-10 I20·0, I21, I22) between April 2015 and April 2018. The patients were identified as STEMI (ICD-10 I21.0-I21.3, I22), NSTEMI (ICD-10 I21.4), MI unknown type (ICD-10 I21.9), and unstable angina (ICD-10 I20.0). We included patients who had their first ACS episode in the study period (index episode) and excluded those who had another admission for ACS in the previous year. Patients with an unknown ACS type initially in their index episodes but with a known ACS type in subsequent episodes within 30 days were reclassified accordingly. ACS patients who underwent coronary revascularization were defined as either percutaneous coronary intervention (PCI, OPCS-4 K49, K50, K75) or coronary artery bypass grafting (CABG, OPCS-4 K40-K46) within 180 days from the index episodes.

### Definition of inequalities used in the analyses

To analyse geographic variation, we used Clinical Commissioning Groups (CCGs) and location of GP practices, which define NHS bodies responsible for planning and commissioning healthcare services for their local area in the UK. If these were missing, the CCGs related to patients’ residences were used. For inequalities defined by demographics, we used HES data on sex, ethnicity, and socioeconomic deprivation quintiles. Ethnicities were defined as White (British, Irish, and other White background), South Asian (Bangladeshi, Indian, and Pakistani), other Asian (Chinese and other Asian background), black (Caribbean, African, and other black background), and mixed/others (mixed and other ethnic background). Socioeconomic status was defined using the area-level Index of Multiple Deprivation (IMD) scores.^19^ IMD quartile was created by dividing the deprivation scores of individual areas into fifths. Each area, and hence every patient within that area, was assigned one of these fifths. Multiple (>2) Long Term Conditions were identified using the Charlson Score,^20^ defined using diagnoses recorded in all hospital admissions within 2 years before the index admission. Frailty syndrome^21^ was defined as the occurrence of one or more of these seven domains within 2 years before the index admission: dementia and delirium, mobility problems, falls and fractures, pressure ulcers and weight loss, incontinence, dependence and care, anxiety, and depression. The list of ICD-10 codes used to define the Charlson Score and the Frailty domains is available in Supplementary material online, *Table S1*.

### Outcomes

The primary outcome was time to all-cause mortality. Mortality was determined using the national death registry. Secondary outcomes were: time-to-revascularization (PCI or CABG surgery), hospitalization for heart failure, bleeding, and stroke/transient ischaemic attack (TIA). Hospitalization outcomes were determined using HES data on primary and secondary diagnoses after index ACS episodes. Follow-up for death and hospital admissions were limited to 31st March 2021.

### Statistical analysis

#### Variation in revascularization by geographical region

The proportion of ACS patients in each CCG who had revascularization procedures within 30 days of admission was calculated. Regional revascularization rates were determined using Funnel plots, including regional standardized ratios calculated by dividing each region's observed number of revascularizations by the predicted number of revascularizations. Logistic regression models were fitted for revascularization adjusting for ACS type, patient characteristics, including age, sex, ethnicity, socioeconomic deprivation quintile, Charlson comorbidity score, frailty status and year of diagnosis, and the interaction terms between ACS type and each of these characteristics. Regions with standardized ratios falling outside the 99.8% confidence bands were highlighted.

#### Effect of sex, ethnicity, and socioeconomic deprivation on revascularization

We used time-to-event analysis to examine the associations of sex, ethnicity, and socioeconomic deprivation with revascularization by fitting a Fine and Gray's proportional sub-distribution hazards model^22^ on the time from ACS episode to revascularization and adjusting for competing risks: age, Charlson score, frailty status, and year of diagnosis.

#### Effect of sex, ethnicity, and socioeconomic deprivation on outcomes

To examine the effect of sex, ethnicity, and socioeconomic deprivation on mortality, we fitted a Cox regression model on time from index ACS episode to mortality adjusted for age, Charlson score, frailty status, and year. This model also included a revascularization indicator as a time-varying covariate to account for patients who had early mortality and were less likely to receive revascularization. To examine the secondary outcomes of heart failure, bleeding, and stroke/TIA hospitalizations, we included ACS patients who had revascularization within 180 days from their index episodes and fitted the Fine and Gray model to examine the effects on mortality of sex, ethnicity, and socioeconomic deprivation adjusted for time from ACS admission to revascularization, patients’ age, Charlson score, frailty status, and year of admission. All models were fitted separately for STEMI, NSTEMI, and unstable angina patients.

##### Ethical considerations

The study received appropriate governance approvals from the University of Leicester Research Ethics Committee and NHS Digital. The study was conducted using anonymized data, and the need for individual consent was waived. The study complied with the Helsinki Declaration. The strengthening the reporting of observational studies in epidemiology guidelines were used for reporting. The lead author is the study guarantor.

##### Role of the funding source

The funders had no role in study design, data collection, analysis, interpretation, or report writing. All authors had full access to all data in the study, and the corresponding author was the final person responsible for deciding to submit for publication.

## Results

### Study population

The analysis included 428 700 ACS patients admitted to NHS hospitals in England between 2015 and 2018. Out of the total, 122 638 (28.6%) had a diagnosis of STEMI, 214 697 (50.1%) had a diagnosis of NSTEMI, 65 136 (15.2%) had a diagnosis of unstable angina, and 26 229 (6.1%) were MI cases of unknown type (*Table 1* and Supplementary material online, *Table S2*). The average age of ACS patients was 70 years, and 36% were female. Ninety percent were of white ethnicity, 6% were of South Asian, 2% were of other Asian, and 1% were of black ethnicity. Twenty-one percent of ACS were from the most socioeconomically deprived quintiles, whereas 18% were from the least deprived quintiles.

**Table 1:** Demographics and characteristics of ACS patients in England 2015–2018

	STEMI	NSTEMI	MI-unknown	Unstable angina	ACS
Number of patients	122 638	214 697	26 229	65 136	428 700
% in ACS	28.6%	50.1%	6.1%	15.2%	100%
Number of patients by year					
2015	31 851	52 056	7790	16 231	107 928
2016	30 370	54 588	5932	16 019	106 909
2017	30 798	54 641	6196	16 110	107 745
2018	29 619	53 412	6311	16 776	106 118
Age, mean (SD)	67 (14.0)	72 (13.6)	76 (13.1)	69 (12.4)	70 (13.8)
Age 15–39	2552 (2%)	2102 (1%)	289 (1%)	522 (1%)	5465 (1%)
40–49	11 684 (10%)	11 564 (5%)	757 (3%)	3758 (6%)	27 763 (6%)
50–59	26 223 (21%)	29 992 (14%)	1996 (8%)	11 719 (18%)	69 930 (16%)
60–69	29 676 (24%)	42 885 (20%)	3882 (15%)	16 586 (25%)	93 029 (22%)
70–79	26 992 (22%)	55 667 (26%)	6690 (26%)	18 447 (28%)	107 796 (25%)
80+	25 511 (21%)	72 487 (34%)	12 615 (48%)	14 104 (22%)	124 717 (29%)
Sex					
Male	84 915 (69%)	132 605 (62%)	14 596 (56%)	40 509 (62%)	272 625 (64%)
Female	37 702 (31%)	82 075 (38%)	11 627 (44%)	24 626 (38%)	156 030 (36%)
Ethnic background					
White	102 837 (90%)	185 791 (90%)	23 124 (92%)	56 088 (88%)	367 840 (90%)
South Asian	6218 (5%)	11 579 (6%)	949 (4%)	4205 (7%)	22 951 (6%)
Asian others	1942 (2%)	2938 (1%)	270 (1%)	1117 (2%)	6267 (2%)
Black	1332 (1%)	2932 (1%)	346 (1%)	915 (1%)	5525 (1%)
Mixed & others	2557 (2%)	3971 (2%)	408 (2%)	1281 (2%)	8217 (2%)
Socio-economic deprivation quintile					
Q1—most deprived	26 283 (22%)	44 499 (21%)	5828 (22%)	14 129 (22%)	90 739 (21%)
Q2	24 652 (20%)	43 595 (20%)	5422 (21%)	13 295 (21%)	86 964 (20%)
Q3	24 867 (20%)	44 239 (21%)	5244 (20%)	13 622 (21%)	87 972 (21%)
Q4	23 720 (20%)	42 281 (20%)	5103 (20%)	12 467 (19%)	83 571 (20%)
Q5—least deprived	21 821 (18%)	38 273 (18%)	4478 (17%)	11 102 (17%)	75 674 (18%)
Charlson Index	1.3 (1.6)	1.9 (2.1)	2.6 (2.2)	1.8 (1.9)	1.8 (2.0)
Frailty syndrome	30 375 (25%)	77 845 (36%)	14 109 (54%)	20 894 (32%)	143 223 (33%)

Expressed as mean (standard deviation) for continuous variables and number (percentage) of patients for categorical variables. Missing data were identified in 45 (0.01%) patients for sex, 17 900 (4.2%) patients for ethnicity, and 3780 (0.9%) for socio-economic deprivation index.

People with NSTEMI (average 72 years) and unstable angina (average 69 years) were more likely to be older (62%), female (62%, respectively), have multimorbidities (mean Charlson index of 1.9) and frailty (36%) compared to the STEMI group (mean age 67 years, 69% female, mean Charlson index 1.3, and frailty syndrome in 25%) (*Table 1*).

### Time to coronary revascularization following ACS diagnosis

**Figure 1:**
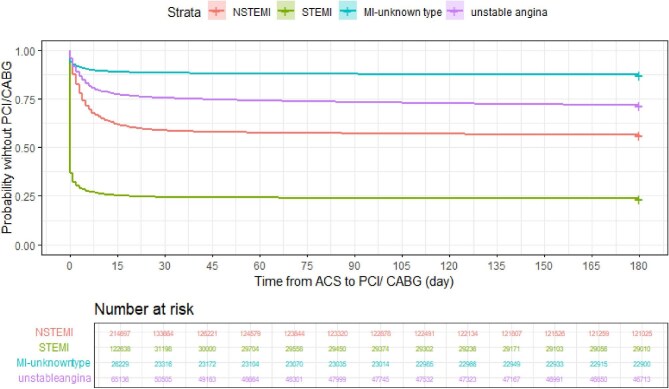
Kaplan–Meier curves showing the revascularization rates based on diagnosis at presentation on a time to event at 180 days analysis.

From all ACS patients, 212 015 (48.8%) were revascularized with PCI or CABG within 30 days of presentation to the hospital (*Figure 1*), including 76.3% of STEMI patients, 43.6% for NSTEMI and 28.3% for unstable angina patients. Of these, 67.9% of STEMI patients underwent revascularization within 1 day of ACS admission, compared to 12.3% for NSTEMI and 8.1% for unstable angina.

**Figure 2:**
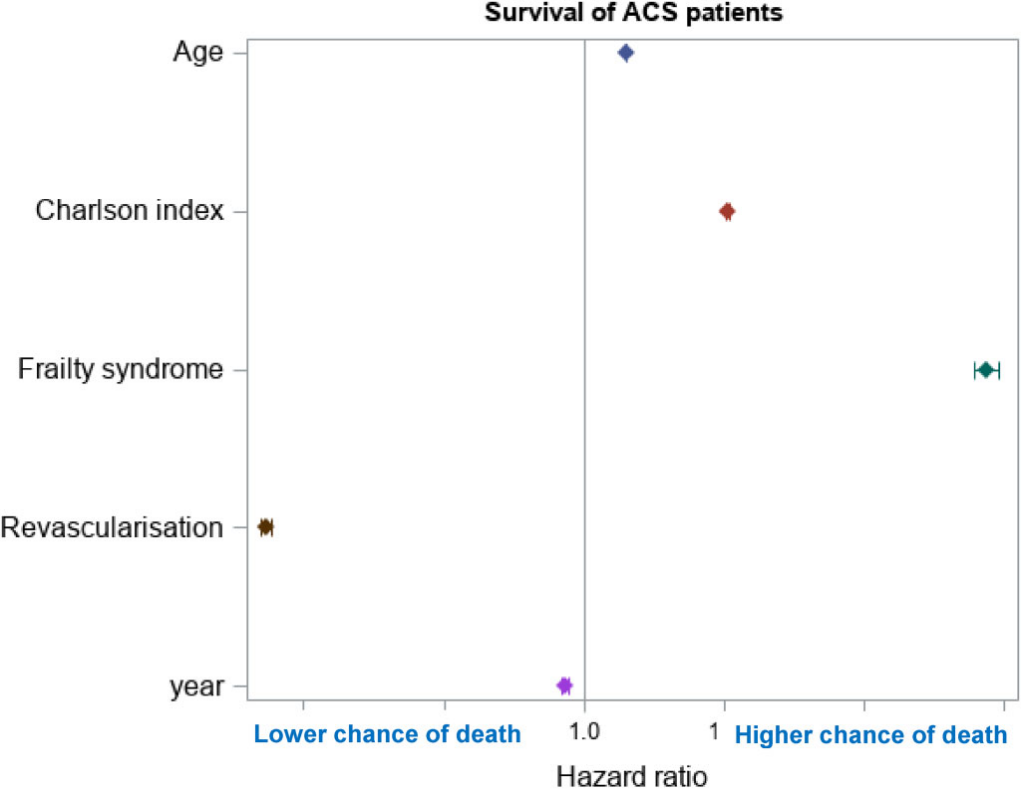
Survival univariate analysis at 1 year based on age, Charlson index, frailty, or revascularization in ACS patients.

The crude proportions of females undergoing revascularization within 30 days from ACS presentation were lower than that of males for all ACS types (*Table 2*). Higher Charlson Index and Frailty scores were also associated with lower hazard ratios (HRs) for revascularization (*Figure 2*).

**Table 2:** Revascularization and outcomes of STEMI, NSTEMI, and unstable angina patients by sex, ethnicity, and socio-economic deprivation quintile.

	STEMI	NSTEMI	Unstable angina
Number of patients	122 638	214 697	65 136
Number of patients who received coronary revascularization (PCI/CABG)	93 628	93 688	18 430
% receiving revascularization	76.3%	43.6%	28.3%
1-year mortality	18.0%	21.1%	7.4%
1-year cardiovascular mortality	16.1%	17.4%	5.4%
In those who received revascularization			
1-year admission for heart failure	11.3%	10.1%	7.6%
1-year admission for bleeding	2.7%	3.0%	2.8%
1-year admission for stroke/TIA	1.5%	1.8%	1.8%
**Revascularization in ACS**			
Overall	76.3%	43.6%	28.3%
By sex			
Male	82.2%	52.5%	33.7%
Female	63.1%	29.4%	19.4%
Ethnic background			
White	75.3%	42.4%	27.8%
South Asian	83.9%	54.8%	32.1%
Other Asian	84.0%	56.6%	34.1%
Black	73.3%	37.0%	19.7%
Mixed & others	82.9%	51.7%	30.8%
Socio-economic deprivation quintile			
Q1—most deprived	76.6%	43.2%	23.5%
Q2	76.5%	43.1%	26.4%
Q3	76.5%	43.8%	28.9%
Q4	75.7%	44.1%	31.5%
Q5—least deprived	76.6%	44.0%	32.4%
**1-year mortality in ACS**			
Overall	18.0%	21.1%	7.4%
Sex			
Male	14.7%	18.7%	7.2%
Female	25.5%	24.9%	7.7%
Ethnic background			
White	18.4%	21.8%	7.8%
South Asian	12.7%	14.9%	4.6%
Other Asian	12.4%	14.3%	3.8%
Black	16.7%	16.5%	6.4%
Mixed & others	12.2%	14.8%	4.1%
Socio-economic deprivation quintile			
Q1—most deprived	17.7%	20.9%	7.3%
Q2	18.4%	21.6%	7.9%
Q3	17.7%	21.4%	7.3%
Q4	18.5%	21.2%	7.2%
Q5—least deprived	17.9%	20.6%	7.3%
**1-year cardiovascular mortality**			
Overall	16.1%	17.4%	5.4%
Sex			
Male	13.2%	15.6%	5.4%
Female	22.6%	20.3%	5.4%
Ethnic background			
White	16.4%	18.0%	5.7%
South Asian	11.6%	13.1%	3.5%
Other Asian	11.0%	12.6%	3.2%
Black	15.0%	14.0%	4.4%
Mixed & others	10.4%	12.2%	3.5%
Socio-economic deprivation quintile			
Q1—most deprived	15.8%	17.1%	5.5%
Q2	16.4%	17.8%	5.6%
Q3	16.0%	17.8%	5.3%
Q4	16.5%	17.6%	5.3%
Q5—least deprived	16.1%	17.1%	5.4%
**1-year admission for heart failure in those who received revascularization**			
Overall	11.3%	10.1%	7.6%
Sex			
Male	10.5%	9.4%	7.4%
Female	13.4%	12.2%	8.1%
Ethnic background			
White	11.6%	10.1%	7.8%
South Asian	13.8%	14.0%	7.5%
Other Asian	12.3%	11.8%	9.4%
Black	13.9%	14.5%	8.9%
Mixed & others	10.8%	8.6%	7.4%
Socio-economic deprivation quintile			
Q1—most deprived	12.3%	11.3%	9.4%
Q2	11.9%	10.7%	8.1%
Q3	11.1%	10.0%	7.4%
Q4	10.8%	9.4%	6.6%
Q5—least deprived	10.4%	9.5%	7.0%
**1-year admission for bleeding in those who received revascularization**			
Overall	2.7%	3.0%	2.8%
Sex			
Male	2.5%	2.9%	2.9%
Female	3.4%	3.4%	2.4%
Ethnic background			
White	2.9%	3.1%	2.9%
South Asian	2.7%	3.2%	2.7%
Other Asian	2.5%	2.0%	2.4%
Black	3.6%	4.1%	0.6%
Mixed & others	3.0%	3.4%	2.3%
Socio-economic deprivation quintile			
Q1—most deprived	2.9%	3.0%	3.1%
Q2	2.8%	3.2%	2.7%
Q3	2.7%	2.9%	2.6%
Q4	2.8%	3.0%	2.7%
Q5—least deprived	2.6%	3.0%	2.9%
**1-year admission for stroke/TIA in those who received revascularization**			
Overall	1.5%	1.8%	1.8%
Sex			
Male	1.3%	1.7%	1.8%
Female	1.9%	2.2%	1.7%
Ethnic background			
White	1.5%	1.9%	1.8%
South Asian	1.6%	1.9%	1.3%
Other Asian	2.2%	2.2%	1.8%
Black	2.0%	2.9%	2.2%
Mixed & others	1.8%	2.2%	2.0%
Socio-economic deprivation quintile			
Q1—most deprived	1.5%	2.0%	2.1%
Q2	1.5%	2.0%	1.7%
Q3	1.4%	1.8%	1.9%
4	1.6%	1.7%	1.7%
Q5—least deprived	1.4%	1.6%	1.5%

**Figure 3:**
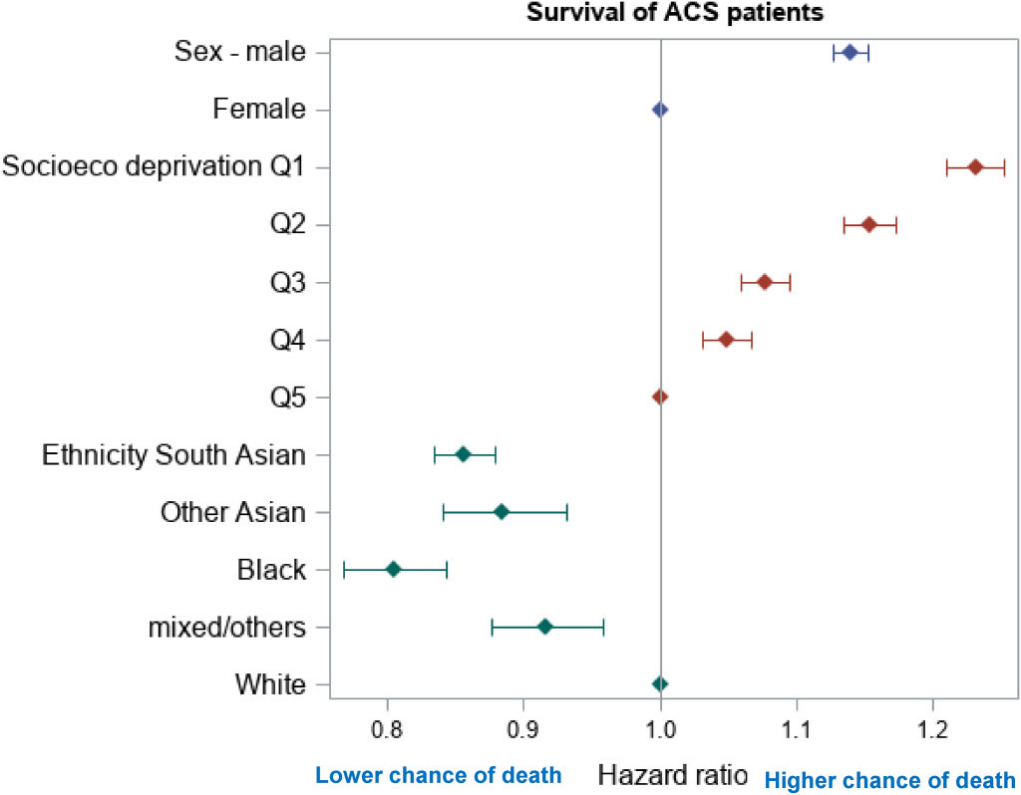
Survival univariate analysis at 1 year based on age, Charlson index, frailty, or revascularization in ACS patients.

After adjustment for other measured sources of inequality, women compared to men remained significantly less likely to receive coronary revascularization [STEMI—HR = 0.86, 95% CI 0.85–0.87, NSTEMI—HR = 0.63, 95% confidence interval (CI) 0.62–0.64, unstable angina—HR = 0.59, 95% CI 0.57–0.61] (*Figure 3*). Across ethnicities, after adjustment, South Asians and other Asians were significantly more likely than whites to undergo revascularization following ACS diagnosis (HR = 1.15, 95% CI 1.14–1.17). People of black ethnicity were less likely to receive revascularization (HR = 0.91, 95% CI 0.87–0.96). The likelihood of revascularization decreased with increasing deprivation quartile (*Figure 3*).

### Geographical variation in revascularization rates

The proportion of ACS patients who had coronary revascularization within 30 days of presentation ranged from 31.7 to 66.8% (mean 47%) between CCGs in England. Funnel plot analysis of observed vs. expected revascularization rates standardized for ACS type, age, sex, ethnicity, and socioeconomic deprivation quintile demonstrated unwarranted variation with 9 of 196 (4.6%) regions’ revascularization rates falling outside the 99.8% CIs (Supplementary material online, *Figure S1A*). Unwarranted variation in revascularization for STEMI was less than for NSTEMI and unstable angina (Supplementary material online, *Figure S1B–S1D*).

### Time to all-cause mortality

Crude 1-year mortality for ACS patients was 18.0% for STEMI, 21.1% for NSTEMI, and 7.4% for unstable angina (*Table 2*). In the univariate analysis, the highest hazard for death at 1 year was frailty (HR 1.6, 95% CI 1.58–1.63), whereas the strongest predictor of survival was revascularization (HR 0.57 95% CI 0.56–0.58) (*Figure 2*). Cardiovascular 1-year mortality was 16.1% for STEMI, 17.4% for NSTEMI and 5.4% for unstable angina (*Table 2*).

Women with ACS had higher crude 1-year mortality compared to men (25.5% vs. 14.7% for STEMI; 24.9% vs. 18.7% for NSTEMI; and 7.7% vs. 7.2% for unstable angina) (*Figure 3*). Cardiovascular 1-year mortality was higher in women compared to men for STEMI and NSTEMI (22.6% vs. 13.2% for STEMI and 20.3% vs. 15.6% for NSTEMI) (*Table 2*). However, after adjusting for known confounders (ACS type, sex, ethnicity, and socioeconomic deprivation), women had a lower hazard for all-cause death vs. men (HR = 0.94, 95% CI 0.92–0.96 for STEMI; HR = 0.84, 95% CI 0.83–0.86 for NSTEMI; and HR = 0.79, 95% CI 0.76–0.82 for unstable angina). Age at presentation was the variable with the largest effect on the adjusted hazard for mortality (Supplementary material online, *Figure S3*). In the same model, people of non-white ethnicity also had lower HRs for mortality vs. Whites (Asians HR = 0.91, 95% CI 0.84–0.97; black HR = 0.74, 95% CI 0.69–0.79; other Asian HR = 0.88, 95%CI 0.83–0.94). Treatment effect estimates were broadly consistent across ACS subtypes (Supplementary material online, *Figure S4*).

Coronary revascularization was associated with significant reductions in time to all-cause death (*Figure 2*). In the final model, after adjustment for Age, Charlson comorbidity, frailty, ethnicity, socioeconomic deprivation, and revascularization, the HR for mortality in Females vs. Males was 0.88 (95% CI 0.87–0.89) (Supplementary material online, *Figure S5*).

### Outcomes in people undergoing revascularization within 180 days of ACS

In adjusted analyses, higher hazards for all-cause death post-revascularization in STEMI, NSTEMI, and unstable angina were observed in males and the most deprived quintiles. People from the most deprived quintiles had higher hazards for rehospitalization for heart failure, bleeding, and stroke TIA across all subgroups. People of South Asian ethnicity had higher hazards of re-admission for heart failure in STEMI and NSTEMI. In contrast, people of black ethnicity had greater hazards for Stoke/TIA when compared to white ethnicity. Blacks also had higher hazards for re-admission for heart failure and bleeding after NSTEMI (Supplementary material online, *Figure S6*).

## Discussion

### Major findings

Lower revascularization rates, worse outcomes following revascularization, and higher all-cause and cardiovascular mortality were observed in women, people of black ethnicity, and people from areas of socioeconomic deprivation. People of South Asian and other Asian ethnicities had higher revascularization rates, equivalent outcomes following revascularization, and lower hazard rates when compared to white ethnicity following ACS.

After adjustment for ACS presentation and measured sources of inequality (age, MLTC, and frailty), females were still associated with lower revascularization rates, but demonstrated improved outcomes following revascularization (lower hazards for 1-year mortality or rehospitalization for heart failure, bleeding, or Stroke/TIA) vs. men and improved risk-adjusted all-cause mortality. South Asians demonstrated consistently better access to revascularization than other ethnic groups. However, outcomes after revascularization were mixed (higher re-admissions with HF after STEMI/NSTEMI, lower mortality at 1-year post-STEMI) compared to white ethnicity. Black ethnicity was associated with lower risk-adjusted revascularization rates and equivalent survival post-revascularization but higher re-admission rates for heart failure, bleeding, and stroke/TIA.

People from quintiles with the highest levels of deprivation demonstrated worse risk-adjusted outcomes across all analyses: revascularization rates, outcomes following revascularization, and overall survival.

#### Clinical importance

Women with ACS demonstrated lower adjusted mortality and better survival after revascularization than men, which is contrary to the direction of effect for unadjusted data. We considered whether bias may have explained these findings. Using a comprehensive national dataset with accurate longitudinal phenotyping, including a revascularization indicator as a time-varying covariate, means that selection bias, lead time bias, or bias by indication are unlikely explanations. These types of bias would also be more likely to have global effects on all estimates. In our analyses of overall mortality, the effects of socioeconomic deprivation and sex were altered by adjustment, whereas the effects of ethnicity were not. Statistically, the difference in unadjusted and adjusted HRs was most likely attributable to the older age of women when compared to men at the ACS presentation. A study evaluating the association between sex and outcomes in 224 249 adults admitted with ACS in Switzerland demonstrated a similar result.^23^ Women may have gender-specific biological and metabolic risks leading to worse outcomes (e.g. premature menopause, endometriosis, and polycystic ovarian syndrome).^24^ Together, these findings suggest that the risks of death and other poor outcomes in women with ACS and who later undergo revascularization are attributable to confounding. Interventions targeting covariates such as age, ethnicity, MLTC, and frailty may be needed to improve outcomes in women.

The second significant finding in our analysis was that social deprivation and black ethnicity negatively impact survival, access to revascularization, and outcomes following revascularization. Multiple unmeasured confounders could explain these findings: effectiveness and compliance with primary and secondary prevention, different ischaemic-bleeding risk profiles, or cardiometabolic burden.^25–27^

The unwarranted geographic variation in revascularization also points to service configuration as an important cause of inequality, disproportionately affecting economically deprived people.^28^ Unwarranted variation of care was lowest for STEMI patients, which also highlights the need for better evidence-based care pathways in NSTEMI.^29^

Despite being affected by earlier and more severe coronary artery disease, South Asians improved their prognosis in other studies, irrespective of the presence of diabetes. This may differ based on country and living conditions-related risk factors and is hypothesis-generating.^30^ South Asians are also heterogeneous; e.g. people from Bangladesh and Pakistan experience higher mortality burdens from cardiovascular disease compared to other South Asian groups.^31^ These are important observations because they provide a framework for addressing potential inequalities both within South Asians and between other underserved groups.

#### Strengths and weaknesses

The study has provided new information beyond recent analyses of inequalities in ACS.^32,33^ First, this study used a larger sample, enabling a more precise evaluation of multiple sources of inequality across multiple ethnic subgroups and clinical presentations, including unstable angina presentations not included in previous reports. Second, the study provided longer follow-up than earlier reports and enabled the assessment of multiple adverse events over 1 year of follow-up. The study also has limitations. First, the lack of data on the severity of coronary artery disease or the application of primary and secondary prevention interventions (including medication data) must be considered important unmeasured confounders. Second, the analyses did not consider other important underserved groups, such as those living with disabilities, carers, and traveller or migrant communities, as these are not recorded in HES data. Third, HES data does not include primary care data or outcomes. These limitations notwithstanding, this analysis yielded several novel insights, not least the improved risk-adjusted survival in females and South Asians and the need for interventions that address inequalities or subgroup-specific factors. Potential clinical trials of interventions may target barriers in availability and variation of care, subgroup-specific risk factors (deprivation status, frailty, reduction in the multimorbidity burden, diet, non-compliance with primary, and secondary prevention) or gene therapies targetting underlying genetic causes for coronary artery disease.

### Conclusion

Using a large contemporary national dataset and multivariate regression analyses to evaluate the intersectionality of multiple sources of health inequality, we have shown that outcomes attributed to women and people of South Asian ethnicity may be attributable to important confounders, including age, comorbidity, and frailty of presentation. Black ethnicity and social deprivation could be important sources of inequality, as does geography. Together, these findings highlight the unmet need for interventions that address inequalities and may provide potential targets for these interventions.

## Supplementary Material

qcae112_Supplemental_File

## Data Availability

NHS Digital provided the data underlying this article under licence. The data supporting this study's findings are available from the corresponding author upon reasonable request. Raw data may be shared with the permission of NHS Digital.
