# Supplementary material for: Intersectionality of inequalities in revascularization and outcomes for acute coronary syndrome in England: nationwide linked cohort study
Source: Eur Heart J Qual Care Clin Outcomes. 2025 Jan 30;11(6):773–82. doi: 10.1093/ehjqcco/qcae112 (PMC12445677; doi:10.1093/ehjqcco/qcae112)
Supplement: qcae112_Supplemental_File [file qcae112_supplemental_file.docx]

# Intersectionality of Inequalities in Access to Treatment and Outcomes in People Presenting with Acute Coronary Syndrome in England

Marius Roman, Ann Cheng, Florence Y Lai, Hardeep Aujla, Julie Sanders, Jeremy Dowling, Sarah Murray, Mahmoud Loubani, Vijay Kunadian, Chris Gale, Gavin J Murphy.

**Supplementary Appendix**

**Figure 1**. Funnel plots of regional standardised revascularisation ratio by Clinical Commissioning Group (CCG) for (A) ACS patients, (B) STEMI, (C) NSTEMI and (D) unstable angina patients.

| **A** 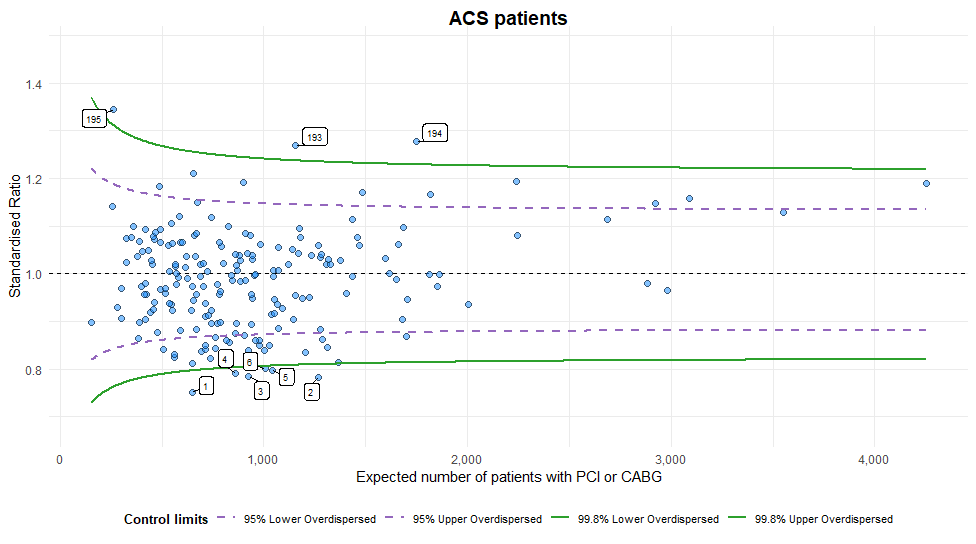 | |  |
| --- | --- | --- |
|  |  |  |

**B**


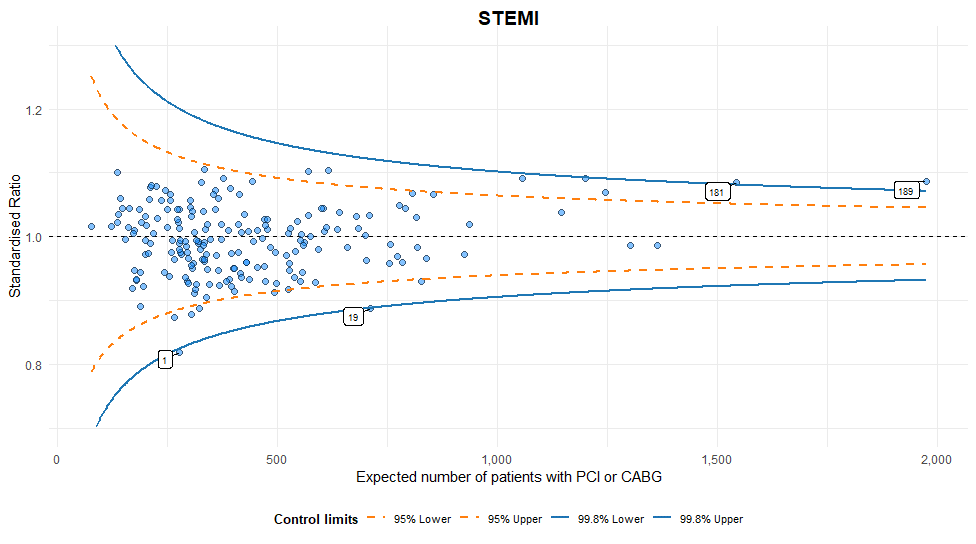


**C**


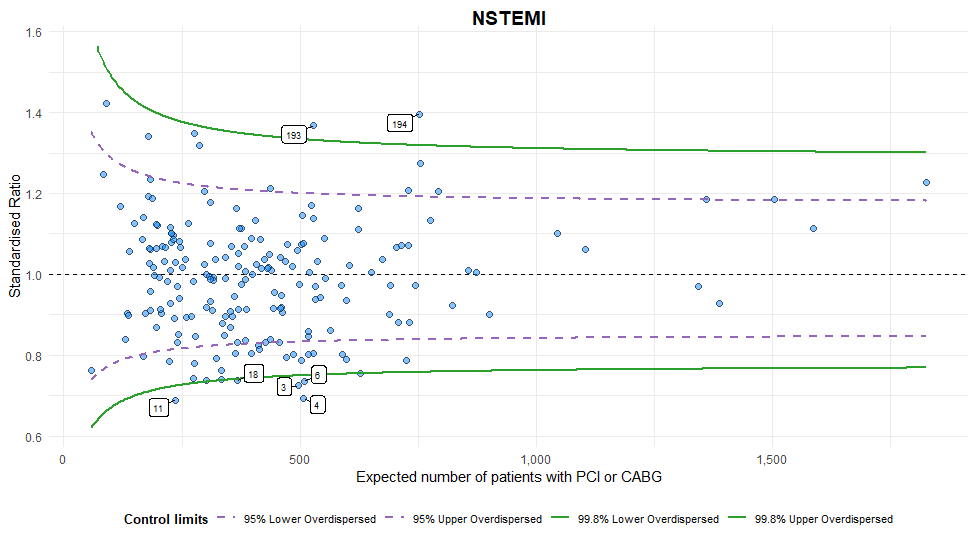


**D**


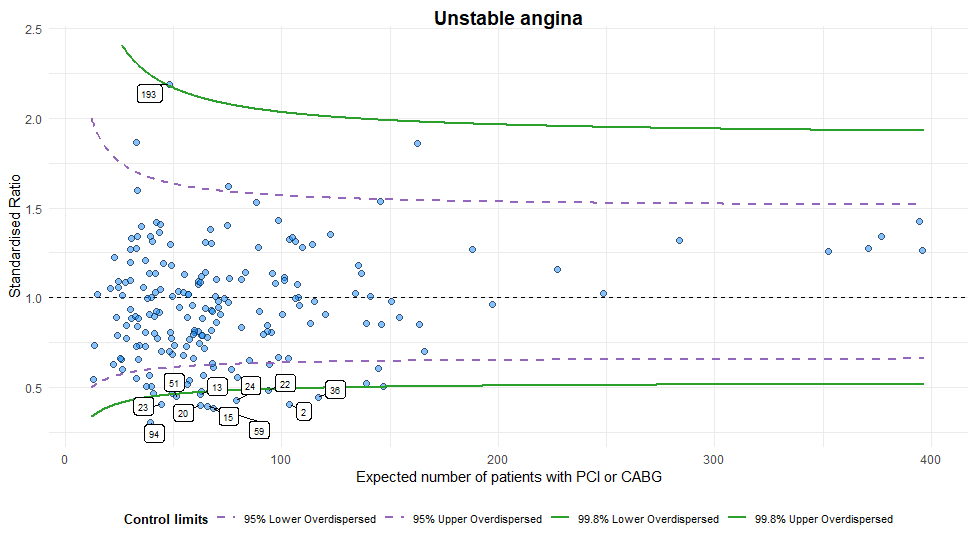


**Supplementary Figure 2.** Crude 1-year mortality, depending on sex.


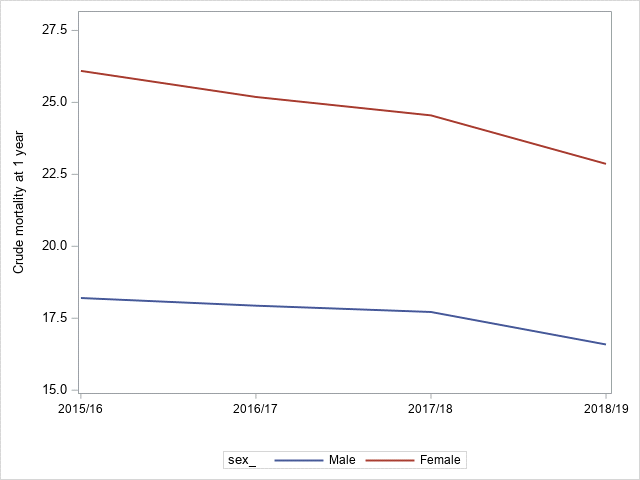


Crude 1-year mortality

**Supplementary Figure 3. A**djusted hazard 1-year mortality for age at presentation.


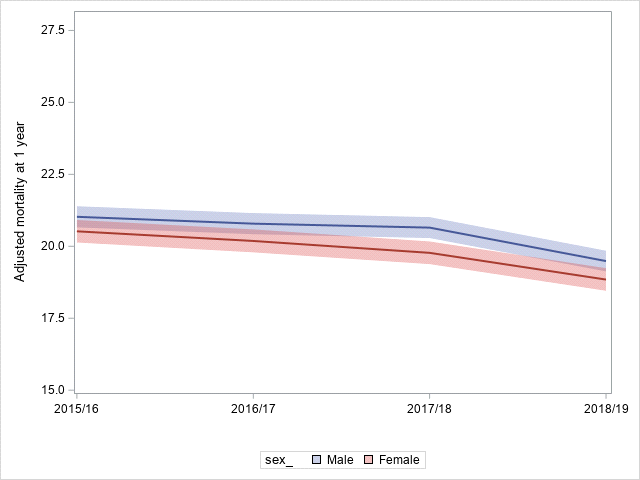


Age-adjusted 1-year mortality

**Supplementary Figure 4**. Time from ACS diagnosis to death univariate analysis in STEMI, NSTEMI and unstable angina patients.


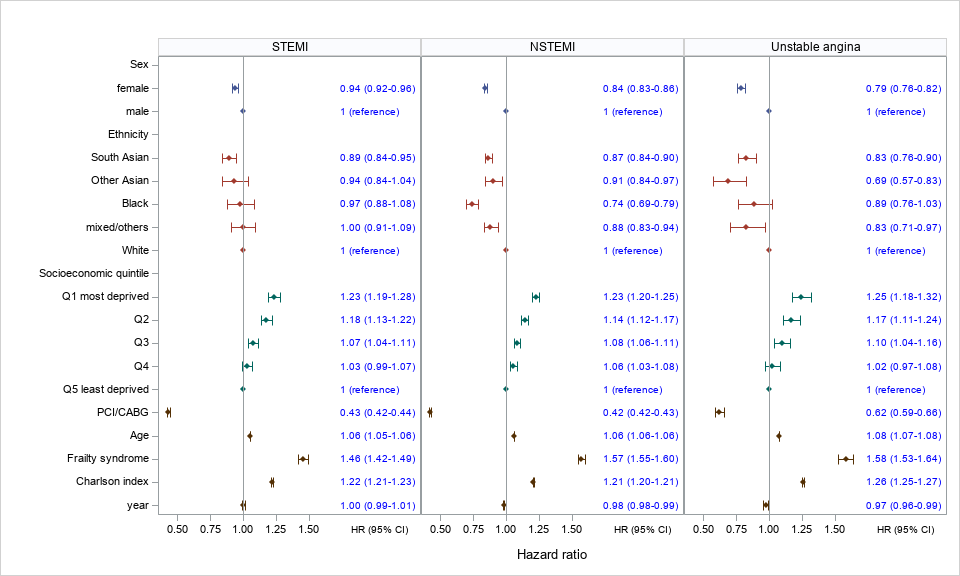


**Supplementary Figure 5.** Association of sex, ethnicity and socio-economic deprivation status on time from ACS diagnosis to coronary revascularisation in STEMI, NSTEMI and unstable angina patients. Models adjusted for patients' age, Charlson comorbidities index, frailty and year of admission.


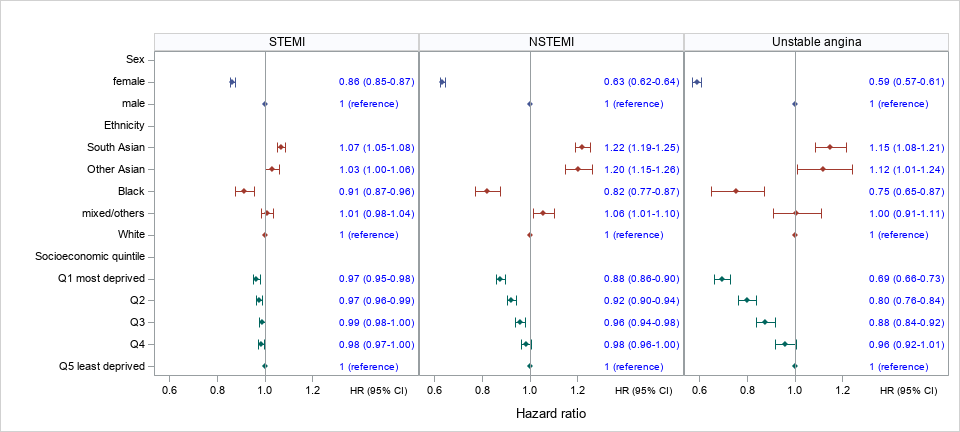


**Supplementary Figure 6.** Association of sex, ethnicity and socio-economic deprivation status with hospital admission for (A) Heart Failure, (B) Bleeding, (C) Stroke/TIA after coronary revascularisation in STEMI, NSTEMI and unstable angina patients. Models adjusted for time from ACS admission to coronary revascularisation, age, Charlson comorbidities index, frailty and year of admission.


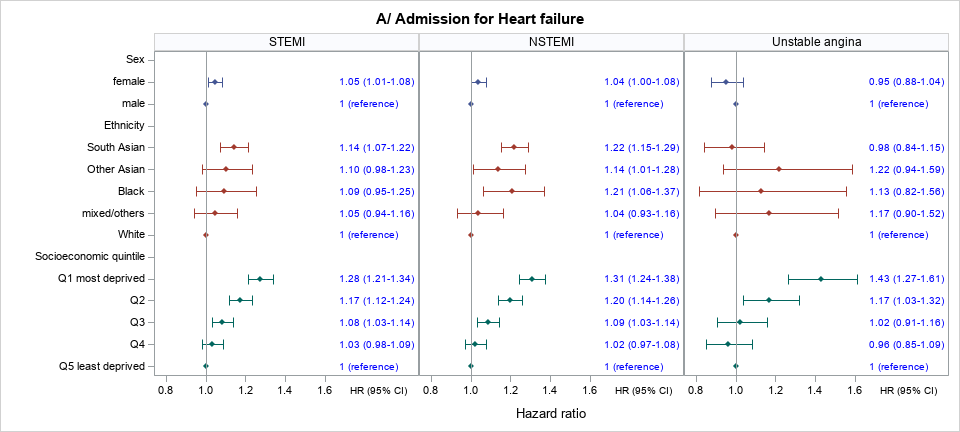

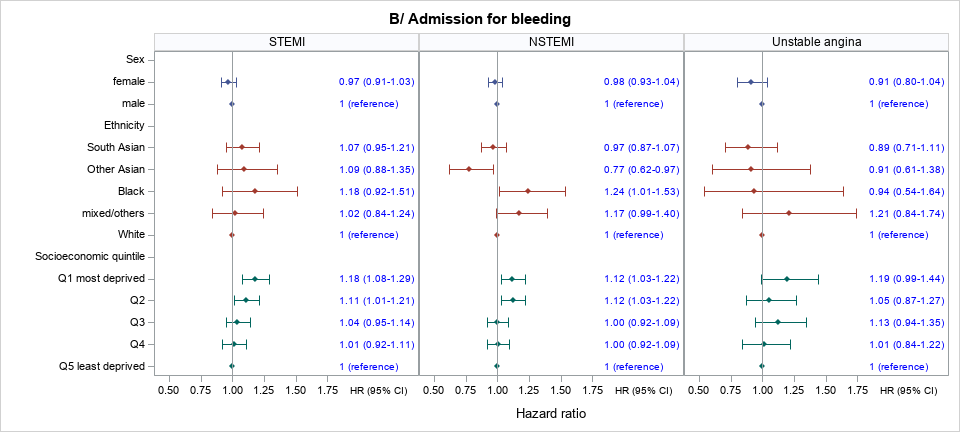

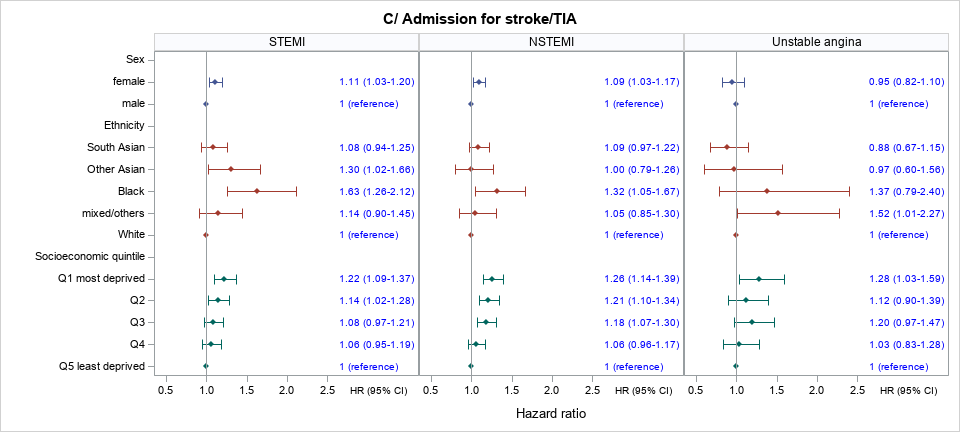


**Supplementary Table 1.** Associated of sex with time to death based on multiple adjusted risk factors: age, Charlson comorbidity index, frailty, ethnicity, year of diagnosis, socio-economic deprivation quartile, and time to revascularisation.

| **Adjustment** | **Sex** | **Hazard ratio (95% CI)** |
| --- | --- | --- |
| unadjusted | M vs F | 0.67 (0.66 - 0.67) |
| Age | M vs F | 1.06 (1.05 - 1.07) |
| Age, Charlson, frailty | M vs F | 1.07 (1.06 - 1.08) |
| Age, Charlson, frailty, ethnicity | M vs F | 1.07 (1.06 - 1.08) |
| Age, Charlson, frailty, ethnicity, IMD | M vs F | 1.08 (1.07 - 1.09) |
| Age, Charlson, frailty, ethnicity, IMD, year | M vs F | 1.08 (1.07 - 1.09) |
| Age, Charlson, frailty, ethnicity, IMD, year, revascularisation* M vs F | | 1.14 (1.13 - 1.15) |

*time dependent covariate

**Supplementary Table 2.** Codes used to define ACS, patient status and outcomes

|  | | |  |
| --- | --- | --- | --- |
| **ACS** | ICD10 | I20.0, I21, I22 |  |
| STEMI | ICD10 | I21.0, I21.1, I21.2, I21.3, I22 |  |
| NSTEMI | ICD10 | I21.4 |  |
| Unstable angina | ICD10 | I20.0 |  |
| **Charlson index** |  |  | Weight |
| Cancer | ICD10 | C00-C76, C81-C85, C883, C887, C889, C900, C901, C91, C92, C93, C940, C941, C942, C943, C945, C947, C95, C96 | 2 |
| Metastatic cancer | ICD10 | C77, C78, C79 | 3 |
| Connective tissue disorder | ICD10 | M05, M060, M063, M069, M32, M332, M34, M353 | 1 |
| Cerebral vascular accident | ICD10 | G450, G451, G452, G454, G458, G459, G46, I60, I61, I62, I63, I64, I65, I66, I670, I671, I672, I674, I675, I676, I677, I678, I679, I681, I682, I688, I69 | 1 |
| Dementia | ICD10 | F00, F01, F02, F051 | 1 |
| Diabetes with long-term complications | ICD10 | E102, E103, E104, E112, E113, E114, E132, E133, E134, E142, E143, E144 | 2 |
| Diabetes without long-term complications | ICD10 | E101, E105, E109, E111, E115, E119, E131, E135, E139, E141, E145, E149 | 1 |
| Congestive heart failure | ICD10 | I50 | 1 |
| HIV | ICD10 | B20, B21, B22, B23, B24 | 6 |
| Mild or moderate liver disease | ICD10 | K702, K703, K717, K73, K740, K742, K743, K744, K745, K746 | 1 |
| Severe liver disease | ICD10 | K721, K729, K766, K767 | 3 |
| Pulmonary disease | ICD10 | J40, J41, J42, J43, J44, J45, J46, J47, J60, J61, J62, J63, J64, J65, J66, J67 | 1 |
| Acute myocardial infarction | ICD10 | I21, I22, I252 | 1 |
| Paraplegia | ICD10 | G041, G81, G820, G821, G822 | 2 |
| Peptic ulcer | ICD10 | K25, K26, K27, K28 | 1 |
| Peripheral vascular disease | ICD10 | I71, I739, I790, R02, Z958, Z959 | 1 |
| Renal disease | ICD10 | N01, N03, N052, N053, N054, N055, N056, N072, N073, N074, N18, N19, N25 | 2 |
| **Frailty syndrome** |  |  |  |
| Dementia and delirium | ICD10 | F00-F05, G30, G311, G310, R41 |  |
| Mobility problems | ICD10 | R26, R298 |  |
| Fall and fractures | ICD10 | S32, S33, S42, S43, S52, S53, S62, S63, S72, S73, W00-W19, M80, M81, M966, R296, R54, R55 |  |
| Pressure ulcers and weight loss | ICD10 | L89, R634, R636, Z724 |  |
| Incontinence | ICD10 | R32, R15 |  |
| Dependence and care | ICD10 | Z74, Z75 |  |
| Anxiety and depression | ICD10 | F204, F251, F31, F064, F32, F33, F38, F41, F43, F44,F341, F412, F432 |  |
| **Outcomes** |  |  |  |
| Heart failure | ICD10 | I50 |  |
| Bleeding | ICD10 | (GI bleed) I85.0, K25.0, K25.2, K25.4, K25.6, K26.0, K26.2, K26.4, K26.6, K27.0, K27.2, K27.4, K27.6, K28.0, K28.2, K28.4, K28.6, K29.0, K62.5, K66.1, K92.0, K92.1, K92.2 (Intracranial bleed) I60 , I61, I62, I69.0, I69.1, I69.2, S06.4 |  |
| Stroke/ TIA | ICD10 | (stroke) I60-I66 , (TIA) G45 |  |
| PCI | OPCS4 | K49, K50, K75 |  |
| CABG | OPCS4 | K40 - K46 |  |

| **Supplementary Table 3.** Components of the Charlson Index and Frailty Syndrome classified by the type of ACS. | | | | | | |  |
| --- | --- | --- | --- | --- | --- | --- | --- |
|  |  |  |  |  |  | |  |
|  | **STEMI** | **NSTEMI** | **MI-unknown** | **Unstable angina** | | **ACS** | |
| Number of patients | 122,638 | 214,697 | 26,229 | 65,136 | | 428,700 | |
| **Charlson index** |  |  |  |  | |  | |
| Cancer | 6% | 9% | 13% | 8% | | 8% | |
| Connective tissue disorder | 3% | 5% | 6% | 5% | | 5% | |
| Cerebral vascular accident | 6% | 10% | 16% | 7% | | 9% | |
| Dementia | 2% | 3% | 6% | 2% | | 3% | |
| Diabetes | 21% | 30% | 31% | 31% | | 28% | |
| Congestive heart failure | 24% | 24% | 35% | 15% | | 24% | |
| Liver disease | 0.5% | 0.8% | 1.8% | 0.7% | | 0.8% | |
| Pulmonary disease | 17% | 24% | 29% | 27% | | 23% | |
| Acute myocardial infarction | 6% | 13% | 15% | 23% | | 13% | |
| Paraplegia | 1% | 2% | 3% | 1% | | 2% | |
| Peptic ulcer | 1% | 2% | 2% | 2% | | 2% | |
| Peripheral vascular disease | 6% | 10% | 14% | 10% | | 9% | |
| Renal disease | 9% | 18% | 26% | 15% | | 16% | |
| **Frailty syndrome** |  |  |  |  | |  | |
| Any one of the below | 25% | 36% | 54% | 32% | | 33% | |
| Dementia and delirium | 8% | 12% | 22% | 6% | | 10% | |
| Mobility problems | 4% | 7% | 12% | 5% | | 6% | |
| Fall and fractures | 12% | 20% | 34% | 15% | | 18% | |
| Pressure ulcers and weight loss | 3% | 5% | 10% | 3% | | 5% | |
| Incontinence | 1% | 2% | 5% | 1% | | 2% | |
| Dependence and care | 1% | 2% | 3% | 1% | | 2% | |
| Anxiety and depression | 9% | 11% | 12% | 15% | | 11% | |
